# Beta Testing an mHealth Symptom Management Intervention for People With Advanced Cancer: Single-Cohort Feasibility and Acceptability Study

**DOI:** 10.2196/94676

**Published:** 2026-09-16

**Authors:** Sharla Wells-Di Gregorio, Tanya Smit, Sarah Baltimore, Don M Benson, Steve K Clinton, Ashley P Davenport, Kari L Kendra, Carolyn J Presley, Donald R Marks

**Affiliations:** 1 Department of Internal Medicine, Division of Palliative Medicine, The Ohio State University Wexner Medical Center, 504 McCampbell Hall North, 1581 Dodd Drive, Columbus, OH, 43201, United States, 1 614-293-8724; 2 Center for Research Excellence in Supportive Care (CREST), The Ohio State University Comprehensive Cancer Center – Arthur G. James Cancer Hospital and Richard J. Solove Research Institute, Columbus, OH, United States; 3 Department of Internal Medicine, Division of Hematology, The Ohio State University Wexner Medical Center, Columbus, OH, United States; 4 The Ohio State University Comprehensive Cancer Center – Arthur G. James Cancer Hospital and Richard J. Solove Research Institute, Columbus, OH, United States; 5 Department of Internal Medicine, Division of Medical Oncology, The Ohio State University Wexner Medical Center, Columbus, OH, United States; 6 Department of Advanced Studies in Psychology, Kean University, Union, NJ, United States

**Keywords:** cancer, mobile health, mHealth, symptom management, mobile app, sleep, anxiety, fatigue, depression, uncertainty

## Abstract

**Background:**

People living with advanced cancer experience more frequent and severe symptoms than those with early-stage disease. Common, distressing symptoms include sleep difficulties, worry and uncertainty, fatigue, and depression. Cognitive behavioral therapy (CBT) and acceptance and commitment therapy (ACT) effectively manage these symptoms but are often too time intensive for patients with multiple appointments, limited energy, and competing priorities. Brief, mobile health (mHealth) interventions provide an accessible alternative, particularly for rural patients with limited access to palliative or psychosocial oncology services.

**Objective:**

Building on our successful in-person and Digital Video Disk–based pilot trial of a 3-session, integrated CBT-ACT symptom management intervention for patients with advanced cancer, Finding Our Center Under Stress (FOCUS), the current study evaluated the feasibility and acceptability of a 4-session mHealth version.

**Methods:**

In this single-cohort feasibility and acceptability study, people with advanced cancer were recruited from hospital-based oncology clinics representing 4 cancer types (breast, melanoma, multiple myeloma, and prostate). Baseline assessments included sociodemographic data, patient-reported outcomes for sleep (Insomnia Severity Index), anxiety (7-item Generalized Anxiety Disorder scale and Penn State Worry Questionnaire), fatigue (Fatigue Symptom Inventory), depression (Center for Epidemiological Studies Depression Scale), and a 7-day sleep diary completed in the app. Participants then completed 4 modules addressing sleep difficulties, worry and uncertainty, fatigue, and depression. Primary outcomes were feasibility and acceptability. Feasibility benchmarks included recruitment (50% enrollment), retention (70%), and app completion (50%). After 6 weeks, participants completed the Internet Evaluation and Utility Questionnaire, and a subset completed qualitative interviews about their experience with the FOCUS app. We report quantitative and qualitative findings and lessons learned from app development.

**Results:**

Overall, 64.7% (11/17) of the approached patients enrolled, and 70% (7/10) completed more than half of the app. Participants rated FOCUS highly for ease of understanding (mean 3.83/4, SD 0.41), convenience (mean 3.67/4, SD 0.82), and utility (mean 3.33/4, SD 0.82). All participants would recommend the app to others and would use it again for future problems. Favorite components included patient videos and the Sleep and Worry and Uncertainty Modules. Suggested improvements included video quality (lighting and sound), sleep diary usability, and additional professional guidance.

**Conclusions:**

The FOCUS intervention was successfully delivered via mobile technology and demonstrated feasibility and acceptability in beta testing. The FOCUS mHealth app offers an evidence-based, accessible symptom management intervention for people with advanced cancer, particularly in rural communities. Guided by participant feedback, FOCUS 2.0 will enhance video segments, add telehealth support, and expand interactive and motivational features. A randomized controlled trial will evaluate its effectiveness.

## Introduction

People with advanced cancer experience more frequent and severe psychological and physical symptoms than people with early-stage cancer [1-4]. Four of the most common and distressing symptoms reported by people with advanced cancer are sleep difficulties, worry and anxiety, depression, and fatigue [1,2,4,5]. These symptoms co-occur and contribute to functional impairment, poorer quality of life, and mortality yet are commonly undertreated, especially in rural communities [6-8]. Effective, accessible symptom management is an essential component of comprehensive survivorship care for people with advanced cancer.

In response to the symptom management needs experienced in advanced cancer, we developed Finding Our Center Under Stress (FOCUS), a brief, integrated cognitive behavioral therapy (CBT) and acceptance and commitment therapy (ACT) symptom management intervention. We targeted sleep, anxiety, fatigue, and depression as these were common and distressing symptoms experienced by patients in our outpatient oncology–palliative medicine clinic [4]. We used the most effective and well-used evidence-based CBT-ACT strategies selected by the principal investigator (PI), who has over 20 years of clinical experience with people with advanced cancer [9-16]. We developed a brief, 3-session intervention that included 2 in-person sessions and 1 Digital Video Disk (DVD) session. The DVD included videos recorded by people with advanced cancer as well as our psychology team. We created a standardized FOCUS patient handbook and a corresponding professional therapy manual that guided the in-person components of the intervention.

Using FOCUS, participants learn skills to self-manage their symptoms and improve their ability to focus on what matters most to them despite these symptoms. We conducted a pilot randomized controlled trial with people with advanced cancer, and the results demonstrated improved sleep on both self-report [17] and physiological measures (ie, actigraphy) [18] and moderate to strong improvements in worry, depression, and fatigue interference. However, in this previous pilot study, the primary reason for noneligibility was transportation (35%), and the primary reasons for study refusal were too many appointments (35%) and being too ill (21%).

To increase access to symptom management, mobile health (mHealth) and telehealth symptom management interventions have been developed. These demonstrate positive effects on self-efficacy, symptom distress, and psychological well-being [19,20]. mHealth is “the use of mobile and wireless devices to improve health outcomes, health care services, and health research” [21]. Positive outcomes have been demonstrated for insomnia [22,23], anxiety [24,25], depression [26-31], and fatigue [25,32]. Meta-analyses indicate that mHealth behavioral interventions impact symptom reduction to an extent comparable to face-to-face interventions [33,34]. However, most of these apps focus on single symptoms, or the studies evaluating them include only people with early-stage cancer after treatment, which may benefit outcomes based on symptom regression to the mean [35,36]. Many interventions are also too lengthy (ie, 12-20 sessions) and do not focus on the specific needs and worries of people with advanced cancer.

This paper describes the translation of our FOCUS symptom management intervention to a brief, 4-module mHealth intervention. We present results of our FOCUS 1.0 beta testing with a small but diverse sample of people with advanced cancer from geographically distinct areas of Ohio. Our goal was to conduct a single-cohort trial to determine whether FOCUS 1.0 was feasible and acceptable to people with advanced cancer and identify next steps to prepare for a randomized clinical effectiveness trial of the FOCUS mHealth intervention.

## Methods

### Design

This was a single-cohort feasibility and acceptability study designed to evaluate trial accrual, app engagement and completion, sleep diary and questionnaire administration, and acceptability. We also completed qualitative interviews to determine strengths and areas for improvement of FOCUS 1.0.

### App Translation Process

The FOCUS 1.0 app was developed between February 2021 and April 2021 based on intervention content from our previous in-person intervention [17]. At this time, there was no multi-symptom app available specifically for people with advanced cancer. Unique features of our app include tailoring to people with advanced cancer, combination of CBT and ACT approaches, and inclusion of a modified exercise program for fatigue. The app was developed pro bono by a local digital health company (Duet Health) in collaboration with the PI (SW-DG), who created the content for the previous in-person clinical trial. App questionnaires and the sleep diary were developed by Duet Health, and the text and video content was entered into the content management system by the PI. Collaborators (DMB, SC, AD, KK, CP, and DM) reviewed the app content, and iterative rounds of feedback were conducted. Materials targeted an average Flesch-Kincaid eighth-grade reading level per Microsoft Word document statistics based on an average Program for the International Assessment of Adult Competencies adult literacy score of 268 in Ohio, corresponding to reading level above the sixth-grade but below a ninth-grade reading level. This reading level was deemed appropriate given our attempt to balance accessibility with fidelity to psychosocial oncology content [37].

### FOCUS 1.0 Intervention

**Figure 1.**
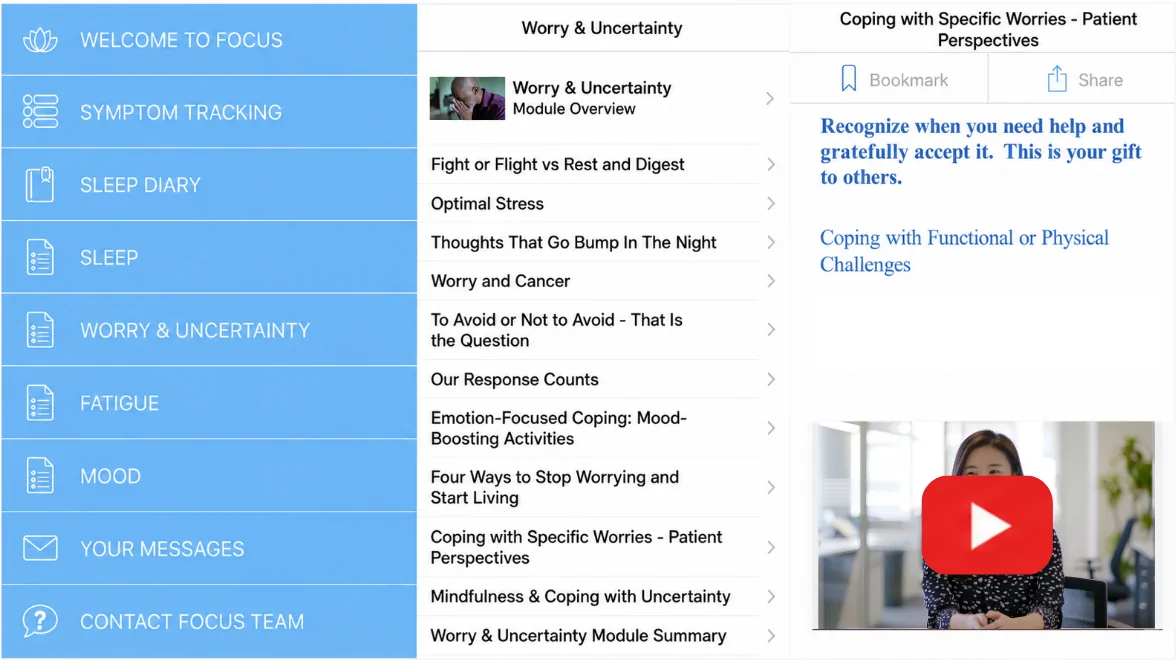
Finding Our Center Under Stress (FOCUS) 1.0 (A) home screen, (B) worry module table of contents, and (C) sample worry intervention content. Original patient video image was replaced by stock photo to protect patient privacy.

FOCUS 1.0 includes 5 total hours of intervention spaced across 6 weeks (not including independent practice of intervention strategies), as well as a 5-minute welcome video, study questionnaires, and information on how to contact the research team. The intervention components of the app are divided into 4 primary modules (Figure 1), each focused on different symptoms as follows: 1.5 hours on sleep, 1 hour on worry and uncertainty, 1.5 hours on fatigue, and 1 hour on mood. The intervention content incorporates CBT and ACT strategies by using text, imagery, videos, and interactive elements throughout the 4 symptom modules. Each module includes informational text and videos from experts to teach participants more about these symptoms and self-management strategies. Modules also include testimonial videos from people living with advanced cancer focused on sharing their experiences with symptom burden, changes in independence, financial stress, and functional challenges and how they have coped with uncertainty. Interactive and instructional relaxation and mindfulness videos are incorporated into the modules, as well as brief exercise videos from our rehabilitation oncology team to target cancer-related fatigue. In addition to the videos, the app incorporates interactive exercises and printable worksheets throughout, including values identification and constructive worry exercises.

### Participants

The current pilot trial included 11 participants recruited from breast cancer, prostate cancer, melanoma, and multiple myeloma clinics based on recommendations from collaborating oncologists. Eligibility criteria were being aged 18 years or older and having a diagnosis of advanced-stage cancer.

### Measures

#### Primary Outcomes

##### Feasibility

We determined feasibility based on potential participants’ willingness to take part in the study, as well as the proportion of intervention modules completed after 6 weeks without prompting. Specifically, feasibility was defined as 50% study enrollment after recruitment (n=17), 70% retention, and completion of at least half (50%) of the app content during the 6-week enrollment period. We also assessed technology skills using the Computer Proficiency Questionnaire−12, a 12-item self-report measure that assesses an individual’s perceived ability to perform common computer tasks across domains such as basic computer operations, internet use, communication, scheduling, and multimedia functions. Respondents rate their proficiency on a 5-point scale, with higher scores indicating greater computer proficiency. The measure has demonstrated excellent internal consistency (Cronbach α=0.95). We used this measure in an attempt to establish a criterion score for use in our larger randomized trial [38].

##### Acceptability

The Internet Evaluation and Utility Questionnaire (IEUQ) [39] measures participants’ experiences and perceptions of an internet-based intervention. The IEUQ has 15 items and 3 open-ended response items. Items 1 to 8 assess ease of use, convenience, engagement, enjoyment, layout, privacy, satisfaction, and acceptability. Items 9 to 15 assess usefulness, comprehension, trustworthiness, credibility, likelihood of returning, mode of delivery, and helpfulness. Participants respond to questions on a 5-point Likert scale (0=“not at all,” 1=“slightly,” 2=“somewhat,” 3*=*“mostly,” and 4=“very”). Scores above 2.5 were considered acceptable. Additionally, the IEUQ includes 3 items that elicit limited qualitative feedback from participants, specifically on what the participants found to be the most and least helpful parts of the app, and provides space for participants to provide suggestions to improve the program. For this study, Cronbach α reliability (items 1-13) was 0.90.

### Secondary Patient-Reported Outcomes

We included several measures to test the viability of assessment of future study patient-reported outcomes (PROs) via the app. These included the Insomnia Severity Index [40], 7-item Generalized Anxiety Disorder scale [41], Fatigue Symptom Inventory [42], Center for Epidemiological Studies Depression Scale [43], the European Organisation for Research and Treatment of Cancer Quality of Life Questionnaire Core 30 [44], and the Penn State Worry Questionnaire [45].

### Procedures

Data collection took place from April 2021 to August 2021. Participants were recruited through collaborating oncology clinics. Each collaborating oncologist was asked to recommend 2 to 3 patients with advanced-stage cancer to beta test the FOCUS app, and they were encouraged to recommend a diverse sample. Potential participants were contacted via phone and were told that their oncologist had recommended them for the study. They were informed that the app was designed to help manage symptoms and cope with cancer and specifically to learn strategies for improving sleep, worry, fatigue, and mood. They were advised that they could participate from home with no in-person appointments. Participants were informed that each module would take between 1 and 2 hours in addition to time to practice the strategies. Interested participants consented to the study via email or mail as per patient preference. Consented participants were then provided with a link to the app, which was available via iOS and Android using their own devices. They were provided a unique password to protect their confidentiality. They completed questionnaires at the start of app use, monitored their sleep for 1 week using the sleep diary feature of the app, and then completed 1 module each week with coping strategies to try. There was no training other than instructions for signing into the app and for completing questionnaires and exercises on the app. Participant instructions (once signed into the app) were as follows: “Complete questionnaires in the Symptom Tracker portion of the app. Then for a week, you will record your sleep for 5 minutes each morning in the Sleep Diary. The following week you will begin the first module and complete one each week starting with the Sleep Module.” The Sleep Module instructions were as follows: “As part of the Sleep Module, you create a sleep restriction schedule based on your data in the Sleep Diary. Go back to your Sleep Diary and review your data for the previous week. Add up the number of hours you slept each night for 7 days and divide by 7. Then add half an hour to this. For instance, if you sleep 6 hours each night: (6 + 6 + 6 + 6 + 6 + 6 + 6)/7 = 6. So you would set your sleep schedule for 6.5 hours with specific wake and bedtimes. Practice this for one week and add 1/2 hour if you are still tired when you wake. And practice relaxation exercises.”

### Ethical Considerations

This study was approved by the Ohio State University Institutional Review Board (2010C0004). All participants were English speakers and provided informed consent via mail or email prior to starting the study.

### Analyses

Given the formative, single-arm nature of this feasibility and acceptability study, our analyses were primarily descriptive rather than inferential. We used percentages to represent feasibility, including the percentage of patients approached for the study who agreed to participate, the percentage who completed the study, and the percentage of modules completed after 6 weeks without prompting. We also report the sample’s computer proficiency scores. Acceptability was examined using descriptive statistics (means and SDs on the IEUQ). Descriptive statistics are also provided on our PROs compared against other cancer sample norms when available, offering an exploratory picture of the symptomatic nature of this population; given the small sample size, these comparisons should be interpreted as hypothesis generating rather than confirmatory. We conclude our analyses with an exploration of our qualitative data from the IEUQ. Content analysis was applied to open-ended items, assessing what was most and least helpful about the app. Two members of the research team independently reviewed and coded participant responses, then conferred to reach consensus on final categories. Given that these items were brief and targeted, designed to capture usability feedback rather than support in-depth qualitative inquiry, this approach was deemed appropriate for the scope and intent of the data. Consistent with the exploratory aim of this study, the findings are therefore presented descriptively rather than as formal themes.

## Results

### Sample Characteristics

A total of 54.5% (6/11) of the sample was female, 72.7% (8/11) were non-Hispanic White individuals, and 72.7% (8/11) were married. The average age was 65.91 (SD 13) years. Participants were diagnosed with breast cancer (4/11, 36.4%), prostate cancer (4/11, 36.4%), melanoma (2/11, 18.2%), and multiple myeloma (1/11, 9.1%), with an average time since diagnosis of 10.55 (SD 8) years. In total, 81.8% (9/11) had prior surgery, 45.5% (5/11) had chemotherapy, 90.9% (10/11) had radiation, 63.6% (7/11) had hormonal therapy, and 18.2% (2/11) had immunotherapy (Table 1).

**Table 1.** Sample demographic and disease characteristics (N=11).

	Values
Sex, n (%)	
Female	6 (54.5)
Male	5 (45.5)
Race, n (%)	
Black	2 (18.2)
Middle Eastern	1 (9.1)
White	8 (72.7)
Age (y), mean (SD)	65.91 (13)
Time since diagnosis (y), mean (SD)	10.55 (8)
Education (y), mean (SD)	15.64 (2)
Marital status, n (%)	
Married	8 (72.7)
Divorced	2 (18.2)
Single	1 (9.1)
Support rating, n (%)	
Limited support	2 (18.2)
Adequate support	5 (45.5)
Excellent support	4 (36.4)
Employment status, n (%)	
Employed full time	1 (9.1)
Retired	7 (63.6)
Disability due to medical condition	2 (18.2)
Other (stay-at-home mother)	1 (9.1)
Financial difficulties, n (%)	
No financial difficulties	10 (90.9)
Yes, limited resources (struggling to pay bills)	1 (9.1)
Yes, limited resources (at risk of losing home)	0 (0)
RUCC^a^, n (%)	
1-3	7 (63.6)
4-7	4 (36.4)
Cancer type, n (%)	
Breast	4 (36.4)
Prostate	4 (36.4)
Melanoma	2 (18.2)
Multiple myeloma	1 (9.1)
Staging, n (%)	
1	1 (9.1)
2	0 (0)
3	2 (18.2)
4	8 (72.7)
Treatments completed, n (%)	
Surgery	9 (81.8)
Radiation	10 (90.9)
Hormonal therapy	7 (63.6)
Chemotherapy	5 (45.5)
Immunotherapy	2 (18.2)
General health (SF-36^b^), n (%)	
Fair	9 (81.8)
Good	1 (9.1)
Very good	1 (9.1)
History of mental health diagnoses, n (%)	
Depression	4 (36.4)
Depression and anxiety	2 (18.2)
Panic attacks	1 (9.1)
Schizoaffective disorder	1 (9.1)

a RUCC: Rural-Urban Continuum Codes.

b SF-36: 36-item Short Form Health Survey.

In total, 36.4% (4/11) were from rural communities (Rural-Urban Continuum Codes 4-9) [46]. Most were retired (7/11, 63.6%), although some were considered disabled due to cancer (2/11, 18.2%), and others still worked full time (1/11, 9.1%). Most (10/11, 90.9%) reported no current financial difficulties related to cancer. A total of 72.7% (8/11) had a history of mental health diagnoses, most commonly depression (4/11, 36.4%) and depression and anxiety (2/11, 18.2%). In total, 9.1% (1/11) had a history of panic attacks, and another participant (1/11, 9.1%) had schizoaffective disorder. Some (2/11, 18.2%) had received psychotropic medications, and some (2/11, 18.2%) had received both medications and counseling or therapy. Most (7/11, 63.6%) had received no mental health treatment despite the high prevalence of mental health diagnoses. Most had adequate (5/11, 45.5%) to excellent (4/11, 36.4%) support.

### FOCUS Feasibility

**Figure 2.**
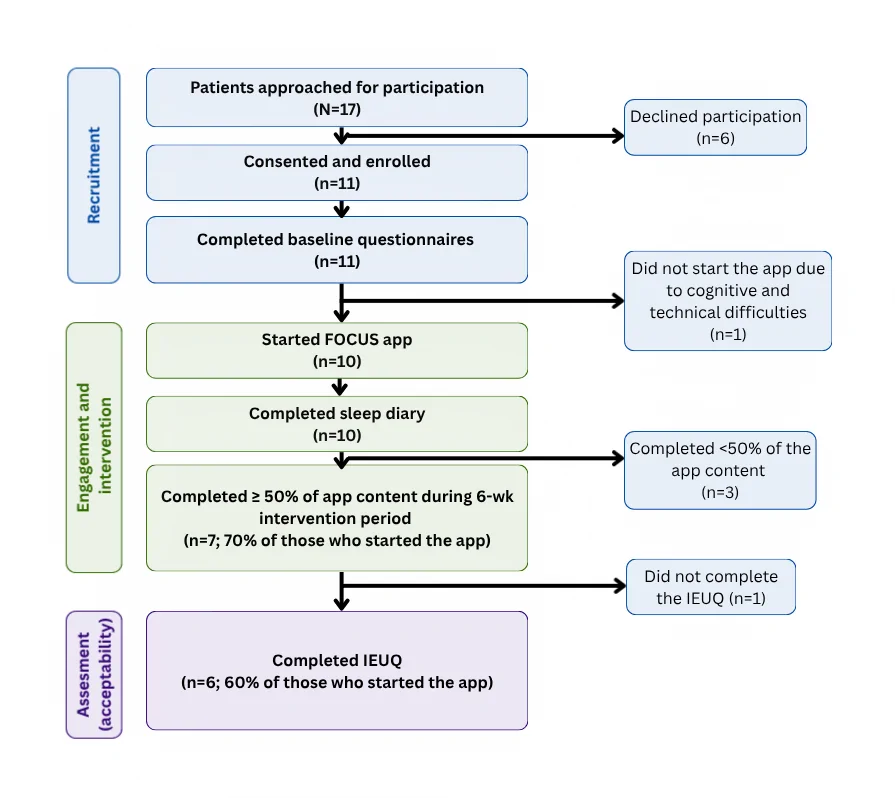
CONSORT (Consolidated Standards of Reporting Trials) flow diagram. FOCUS: Finding Our Center Under Stress; IEUQ: Internet Evaluation and Utility Questionnaire.

Participant flow through FOCUS 1.0 beta testing is shown in Figure 2. Of 17 patients with advanced cancer approached for participation, 11 (64.7%) enrolled and completed the baseline questionnaires. Of these 11 participants, 1 (9.1%) was unable to engage with the intervention because of cognitive and technical difficulties and did not initiate app use, leaving 10 (90.9%) participants who started the FOCUS app and completed the sleep diary. Of these 10 participants, 7 (70%) completed more than 50% of the app content during the 6-week intervention period, meeting the predefined feasibility criterion. Those not completing 50% (of the app) (3/10, 30%) noted challenges with the sleep diary and difficulty navigating the app, and another participant did not start the app due to other family or life circumstances. A total of 60% (6/10) of the participants completed the IEUQ used to assess intervention acceptability.

### FOCUS Acceptability

**Figure 3.**
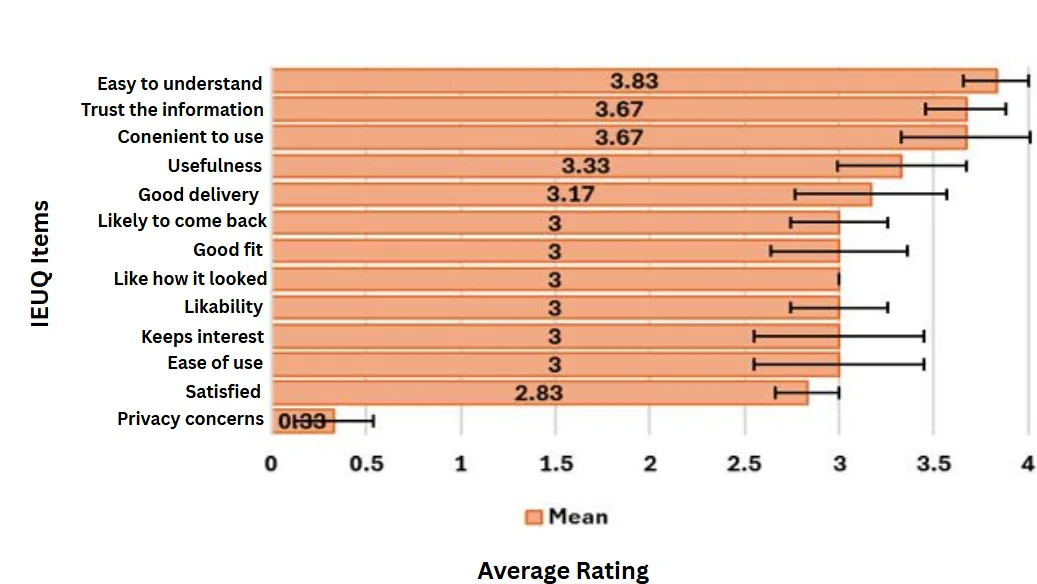
Average Internet Evaluation and Utility Questionnaire (IEUQ) ratings including standard error (n=6). A total of 30% (3/10) of the participants completed <50% of the app after 6 weeks and were excluded as they did not complete the app. Another participant did not complete the IEUQ.

All ratings of the app on the IEUQ were above 3.00 (“mostly” or “very”) other than the satisfaction rating, which was 2.83 (“somewhat” to “mostly”; Figure 3). Participants rated FOCUS highly for ease of understanding (mean 3.83/4, SD 0.41), trust (mean 3.67/4, SD 0.52), convenience (mean 3.67/4, SD 0.82), and utility (mean 3.33/4, SD 0.82).

### PROs

As anticipated, participants were highly symptomatic at baseline given their disease status. Participants scored well above the mean compared to other cancer samples on the Insomnia Severity Index, 7-item Generalized Anxiety Disorder scale, Fatigue Symptom Inventory, Center for Epidemiological Studies Depression Scale, and Penn State Worry Questionnaire (see Table 2). Participants scored below the mean on the European Organisation for Research and Treatment of Cancer Quality of Life Questionnaire Core 30 for cancer samples. Computer proficiency scores were superior compared to older adults in the general population, demonstrating good computer proficiency in this sample [38].

**Table 2.** Patient-reported outcomes (PROs) at baseline.

PROᵃ	Scale range	Cutoff scoresᵇ	Sample, mean (SD)	Cancer populationᶜ, mean (SD)
Insomnia (ISI)	0-28	Mean≥10	11.55 (7.09)	7.3 (6.3)-12.4 (6.7)
Anxiety (GAD–7)	0-21	Mean≥8	6.5 (5.8)	5.1 (4.0)-6.7 (5.2)
Fatigue (FSI)	1-10	Mean≥3	5.0 (2.5)	3.7 (2.2)
Depression (CES-D)	0-60	Mean≥16	19.8 (14.1)	11.6 (9.1)
Quality of life (EORTC-QLQ-C30)	0-100	Mean≤50	39.4 (13.5)	69.3 (20.7)
Worry (PSWQ)	16-80	Mean≥45ᵈ	48 (15.06)	43.3(13.1)
Computer proficiency (CPQ-12)	6-30	10	25.6 (4.1)	NAᵉ

a ISI: Insomnia Severity Index, GAD-7: 7-item Generalized Anxiety Disorder scale, FSI: Fatigue Symptom Inventory, CES-D: Center for Epidemiological Studies Depression Scale, EORTC-QLQ-C30: European Organisation for Research and Treatment of Cancer Quality of Life Questionnaire Core 30, CPQ-12: Computer Proficiency Questionnaire–12.

b Cutoffs: Insomnia Severity Index [47], 7-item Generalized Anxiety Disorder scale [48], Fatigue Symptom Inventory [49], Center for Epidemiological Studies Depression Scale [50], and European Organisation for Research and Treatment of Cancer Quality of Life Questionnaire Core 30 [51]. CPQ-12 scores < 10 are below average compared to the general population and represent users who may need additional training or support [38].

c Cancer population norms: Insomnia Severity Index [40,52], 7-item Generalized Anxiety Disorder scale [53,54], Fatigue Symptom Inventory [55], Center for Epidemiological Studies Depression Scale [56], and European Organisation for Research and Treatment of Cancer Quality of Life Questionnaire Core 30 [57].

d No official cutoff score for the PSWQ exists. The cutoff score of M≥45 is often used to indicate elevated worry [58].

e NA: not available.

### Qualitative Feedback

Qualitative feedback was collected via 3 open-ended items from the IEUQ, which asked participants to identify the most and least helpful aspects of the app and offer suggestions for improvement. Given the targeted nature of these items, the findings are organized below by valence rather than formal thematic analysis.

#### Strengths

All participants would recommend the app to others and would use it again for future problems. Participants’ favorite aspects of the app included the patient videos. One participant noted the following:

I have never participated in support groups as I am an introvert, but seeing that others are experiencing the same thoughts, feelings, and symptoms as me has been a great source of comfort.

Participants preferred the Sleep Module and the Worry and Uncertainty Module. In particular, they appreciated learning about relaxation techniques and sleep restriction, the stretching and relaxing recording (Fatigue Module), and challenging stressful sleep thoughts. One person noted the following:

I would give the top rating to all aspects of the program. I cannot even begin to tell you what it has done for me. I hope that I will be able to always view the information so I can use it in the future if I start feeling down again.

#### Areas for Improvement

Feedback on areas for improvement centered on technical issues, including difficulty accessing the app-based sleep diary and questionnaires; video streaming interruptions and quality issues (ie, audio and lighting); and a desire for access to professional and technical support.

## Discussion

### Primary Findings

In this single-cohort feasibility and acceptability study, participants with advanced cancer evaluated FOCUS 1.0, an integrated mHealth CBT-ACT symptom management intervention targeting sleep, anxiety, fatigue, and depression. FOCUS 1.0 was developed by translating the content of our previous 3-session FOCUS intervention [17] into an mHealth app, which we successfully developed over a period of 3 months. For the current study, we recruited a small but diverse sample, with higher rates of rural (4/11, 36.4%) and minority group (3/11, 27.3%) participants than the rates in our state (24% and 24%, respectively) [59,60]. We also had an impressive recruitment rate (11/17, 64.7%) for a sample of people with advanced cancer [61,62]. We did not specifically include or exclude people with a history of mental health diagnoses and were surprised to find that 72.7% (8/11) reported a history of psychological diagnoses, demonstrating our ability to recruit a sample with diverse mental health concerns. Most participants (7/10, 70%) completed at least 50% of the app content and provided overall positive acceptability ratings. These preliminary findings provide initial support for the feasibility and acceptability of FOCUS 1.0 as an intervention for patients with advanced cancer. Our PRO data are consistent with the highly symptomatic nature of this population of patients who may benefit from the app.

### Participant Feedback

Participants rated ease of understanding very highly, demonstrating that our efforts to enhance readability of content with a sixth to ninth grade Flesch-Kincaid reading level were helpful. Participants also rated convenience and intervention method highly, which is a promising early signal although not conclusive evidence of reach or effectiveness. It is important to note that living with advanced cancer presents many barriers to in-person interventions, including but not limited to travel challenges, difficulty engaging due to feeling ill, and limited time due to having many medical appointments. This intervention attempts to remedy some of these barriers by providing skills and resources in an accessible format. While we cannot draw conclusions on the effectiveness of the intervention with these pilot data, participants found the content useful to address their needs, a criterion essential for motivating app use, particularly among older adults [63,64]. They trusted the content and would revisit it if problems arose again in the future.

Results from qualitative feedback highlighted several strengths of FOCUS 1.0. Participants particularly liked patient-recorded videos designed to share how other patients approach several cancer-related worries. Participants also enjoyed the interactive practice videos, including videos demonstrating relaxation techniques and physical activity to address fatigue (eg, stretching and relaxing). The Worry and Uncertainty and Sleep Modules were reported to be the most helpful. In particular, participants found utility in identifying unhelpful thoughts related to sleep [65,66] and sleep restriction.

### Lessons Learned

While the current results are promising, we encountered several issues that should be noted and addressed in future studies (Table 3). First, participants experienced a variety of technical issues that resulted in understandable frustration. For example, participants noted that the sleep diary designed for the app did not auto-calculate an average time in bed sleeping, which is essential for sleep restriction and scheduling (a primary component of the Sleep Module). To address technical issues, we recommend conducting thorough testing to ensure that the components of the mHealth app are working properly and building in a virtual technical support component to address patient questions and enhance patient motivation for app completion [67-71]. Second, including the questionnaires as the initial step on the app created undue participant burden and limited enthusiasm for venturing further with the symptom modules. Additionally, we encountered a glitch with our questionnaire system such that our week 6 questionnaires were not released to participants automatically, with consequent data loss for this follow-up assessment. To address this, we recommend building the questionnaires outside of the app in a system such as REDCap or Qualtrics. In addition to reducing the perceived burden associated with the mHealth app for participants, this would reduce the workload for researchers associated with privacy protections when data are collected within an app and potentially prevent issues with data loss. Third, participants reported a desire for additional professional support in terms of learning symptom management strategies. To address this, we have developed a telehealth professional support component to improve user motivation and experience using the app. Fourth, 9.1% (1/11) of the recruited participants were unable to engage with the intervention content due to cognitive and technological issues that we could not resolve by providing additional support. Future studies testing mHealth interventions may benefit from incorporation of cognitive and mobile proficiency screening.

**Table 3.** Finding Our Center Under Stress (FOCUS) 1.0 problems and FOCUS 2.0 planned solutions.

FOCUS 1.0 problem	FOCUS 2.0 solution
Sleep diary not working properly to calculate time in bed sleeping	Constructing a new sleep diary that auto-calculates time in bed sleeping for ease of participant use
General technical issues	Grant funding included for technical support to reduce technical barriers; use of mobile and computer proficiency tests to screen participants
Questionnaires built into app and not delivered on schedule	Questionnaires now built outside of the app in REDCap to reduce burden and privacy risks in the app and scheduled for repeat administration
Desire for more professional support	Designed a telehealth professional support protocol and manual to accompany app use
Participants with cognitive impairment	Screening for cognitive impairment as part of the prescreening process

### Limitations

Several limitations should be considered when interpreting the findings of this study. First, the small sample size (N=11) limits generalizability; however, given the feasibility and acceptability focus of this study, the sample size is appropriate for generating initial evidence. Second, qualitative data were collected via brief open-ended items designed to capture user feedback on app usability rather than to support more sophisticated qualitative analysis (eg, thematic analysis). Future studies should incorporate more rigorous qualitative methods to more fully capture participant perspectives and inform iterative intervention refinement. Third, for this study, we relied on self-reported sleep measures and did not include objective monitoring devices. While the focus at this stage of app development was app user experience, including app-based sleep diary development, we recognize this as a limitation and plan to use objective sleep monitoring devices (ie, actigraphy watches) in our larger randomized clinical trial. Finally, despite higher-than-average computer proficiency, at least 30% (3/10) of the participants experienced technical difficulties. This study did not provide formal technical support for participants, which may have influenced engagement and usability outcomes. Addressing these limitations in future iterations will be important for strengthening implementation and evaluation, as well as participant intervention engagement.

### Conclusions

In conclusion, although individuals coping with advanced cancer experience substantial psychological and physical symptom burden, few have access to interventions that effectively support symptom management. Findings from this feasibility and acceptability study suggest that FOCUS 1.0 shows promise in addressing these gaps in care. This study represents an initial, formative step in the iterative process of refining and evaluating the FOCUS intervention. While additional research is needed to establish the efficacy of FOCUS 1.0, this mHealth intervention has the potential to offer an accessible and scalable psychosocial treatment option for patients with advanced cancer, including those living in rural and underserved communities.

## Abbreviations

ACT: acceptance and commitment therapy
CBT: cognitive behavioral therapy
DVD: Digital Video Disk
FOCUS: Finding Our Center Under Stress
IEUQ: Internet Evaluation and Utility Questionnaire
mHealth: mobile health
PI: principal investigator
PRO: patient-reported outcome
